# Intraoperative mechanical ventilation and incidence of pneumothorax in lymphangioleiomyomatosis

**DOI:** 10.1186/s13023-024-03117-w

**Published:** 2024-03-23

**Authors:** Chen Sun, Lijian Pei, Chongsheng Cheng, Bing Bai, Kai-Feng Xu, Yuguang Huang

**Affiliations:** 1 Department of Anesthesiology, Peking Union Medical College Hospital, Chinese Academy of Medical Sciences, Beijing, China; 2 Department of Pulmonary and Critical Care Medicine, Peking Union Medical College Hospital, Chinese Academy of Medical Sciences, Beijing, China; 3 State Key Laboratory of Complex Severe and Rare Diseases, Peking Union Medical College Hospital, Chinese Academy of Medical Sciences, Beijing, China; 4 Outcomes Research Consortium, Cleveland, OH USA

**Keywords:** Intraoperative mechanical ventilation, Lymphangioleiomyomatosis, Pneumothorax

## Abstract

Patients with lymphangioleiomyomatosis (LAM) are considered high risk for most surgeries and require specific anesthetic considerations mainly because of the common spontaneous pneumothorax (PTX). To explore whether intraoperative mechanical ventilation could increase the risk of PTX in those patients, we included 12 surgical patients with LAM in this study, of whom four (33.3%) experienced postoperative PTX. According to our results, patients with higher CT grade, poorer pulmonary function, and a history of preoperative PTX might be more likely to develop postoperative PTX. However, intraoperative mechanical ventilation did not show obvious influence, which might help clinicians reconsider the perioperative management of LAM patients.

**Supplementary Information:**

The online version contains supplementary material available at 10.1186/s13023-024-03117-w.

## Introduction

Lymphangioleiomyomatosis (LAM) is a rare disease affecting almost exclusively women in reproductive age. It is characterized by the proliferation of abnormal smooth muscle-like cells (LAM cells) in the lungs and lymphatic system, and is considered as a low-grade metastasizing neoplasm [1–3]. In the lungs, LAM cell proliferation leads to the development of multiple thin-walled cysts and progressive destruction of the parenchyma, resulting in dyspnea, obstructive ventilatory, reduced carbon monoxide transfer factor, and hypoxemia [4]. Pneumothorax (PTX) is a common manifestation of LAM. Previous studies have demonstrated that approximately 66% of patients with LAM may exhibit pneumothorax; importantly, 70% of these patients may experience recurrent ipsilateral or contralateral pneumothoraces [5]; therefore, these patients are considered high risk for most surgeries and require specific considerations for anesthesia [6]. Several studies, mostly case reports, have discussed perioperative management strategies for LAM patients and recommended regional anesthesia to prevent the rupture of closed lung cysts and further PTX [7]. However, regional anesthesia may be not suitable for all procedures, and patients inevitably need positive pressure ventilation under general anesthesia in certain situations. Considering this, we hope to explore whether intraoperative mechanical ventilation could increase the incidence of PTX in patients with LAM.

## Methods

This study was approved by the Institutional Review Board of Peking Union Medical College Hospital (K2197). We retrieved all the surgical patients diagnosed with LAM in Peking Union Medical College Hospital through Anesthesia Information Management System between January 2013 and June 2022. The diagnosis of LAM was based on the American Thoracic Society and Japanese Respiratory Society guidelines published in 2017 [8]. The primary outcome was new or recurrent PTX within 30 days after surgery (including intraoperative period), confirmed by radiological imaging of the chest. Data including demographics, preoperative variables and intraoperative variables were reviewed. For preoperative variables, the high-resolution computerized tomography (CT) grade of LAM (classified according to the proportion of cystic lesions in the total lung), vascular endothelial growth factor-D (VEGF-D) level, pulmonary function, 6-min walking distance (6MWD), history of PTX, history of pleurodesis, menstruation and pregnancy status, as well as mammalian target of rapamycin (mTOR) inhibitor therapy were analyzed. Among them, high-resolution CT, VEGF-D level, 6MWD, and pulmonary function were all tested within 30 days before surgery. As for intraoperative variables, the surgical type, anesthetic technology, surgical and anesthetic duration and mechanical ventilation were reviewed. Because of the small number of patients involved in this retrospective study, a description of cases was used, while we still tried to conduct the descriptive statistics as an exploratory analysis. A 2-sided *P* value less than 0.05 was considered the threshold for statistical significance.

## Results

We finally included 12 surgical patients with a definite diagnosis of LAM. They were all females with a mean age of 41 ± 10 years, among whom four (33.3%) experienced PTX within 30 days after surgery (three recurrent and one new case, no intraoperative PTX). As shown in Table 1, more patients had high CT grade (III) in postoperative PTX group (PP group) than no postoperative PTX group (nPP group) (66.7% vs. 37.5%). Patients in the PP group showed poorer pulmonary function compared to those in the nPP group (FEV_1_%pred 69.5 ± 7.8% vs. 79.1 ± 28.7%, DLco%pred 36.0 ± 15.6% vs. 66.3 ± 29.9%, PaO_2_ 71.4 ± 10.2 mmHg vs. 84.5 ± 13.3 mmHg, SpO_2_ 93 ± 6% vs. 98 ± 2%). 75% of patients in PP group had a history of spontaneous PTX before surgery, while only 37.5% in nPP group. A total of three patients received preoperative mTOR inhibitor therapy. In one patient, everolimus (5 mg, Qd) was taken for 2.5 months and stopped 15 days before surgery due to the herpes zoster. The second patient had received sirolimus (1 mg, Qd) therapy since 23 days before surgery. The third patient had been treated with sirolimus (1 mg, Qd) for 9 years, and stopped 28 days before surgery. All three patients did not develop postoperative PTX, and two of them received intraoperative mechanical ventilation. However, there is no significant difference between the two groups according to the statistical analysis (Suppl 1).

**Table 1:** Baseline characteristics of surgical patients with lymphangioleiomyomatosis

Variables	All patients (*n* = 12)	PP group (*n* = 4)	nPP group (*n* = 8)
Age (y)	41 ± 10	37 ± 9	43 ± 11
Diagnosis			
TSC-LAM	1 (8.3)	1 (25.0)	0 (0.0)
Sporadic-LAM	11 (91.7)	3 (75.0)	8 (100.0)
**Preoperative variables**			
CT grade^*a*^			
I	5 (45.5)	0 (0.0)	5 (62.5)
II	1 (9.1)	1 (33.3)	0 (0.0)
III	5 (45.5)	2 (66.7)	3 (37.5)
VEGF-D (pg/mL)^*b*^	1145 ± 832	1208 ± 528	1119 ± 971
FEV_1_%pred (%)^*c*^	77.2 ± 25.8	69.5 ± 7.8	79.1 ± 28.7
DLco%pred (%)^*d*^	60.2 ± 29.7	36.0 ± 15.6	66.3 ± 29.9
6MWD (m)^*e*^	501 ± 69	491 ± 91	505 ± 66
PaO_2_ (mmHg)	80.1 ± 13.5	71.4 ± 10.2	84.5 ± 13.3
SpO_2_ (%)	96 ± 4	93 ± 6	98 ± 2
Preoperative PTX	6 (50.0)	3 (75.0)	3 (37.5)
History of pleurodesis	2 (16.7)	0 (0.0)	2 (25.0)
Menstruation status			
Pre-menopausal	9 (75.0)	3 (75.0)	6 (75.0)
Peri-menopausal	1(8.3)	1 (25.0)	0 (0.0)
Post-menopausal	2 (16.7)	0 (0.0)	2 (25.0)
Current pregnancy	4 (33.3)	2 (50.0)	2 (25.0)
Preoperative mTOR inhibitors therapy	3 (25.0)	0 (0.0)	3 (37.5)
**Intraoperative variables**			
Surgical type			
VATS lobectomy or pleurodesis	6 (50.0)	2 (50.0)	4 (50.0)
Partial nephrectomy	1 (8.3)	0 (0.0)	1 (12.5)
Hysterectomy and bilateral salpingoophorectomy	1 (8.3)	0 (0.0)	1 (12.5)
Cesarean section	4 (33.3)	2 (50.0)	2 (25.0)
Surgery duration (min)	79 ± 40	79 ± 28	79 ± 47
Timing of surgery			
Emergent	1 (8.3)	1 (25.0)	0 (0.0)
Elective	11 (91.7)	3 (75.0)	8 (100.0)
ASA physical status			
1	2 (16.7)	0 (0.0)	2 (25.0)
2	5 (41.7)	1 (25.0)	4 (50.0)
3	4 (33.3)	2 (50.0)	2 (25.0)
4	1 (8.3)	1 (25.0)	0 (0.0)
Anesthetic technology			
General anesthesia	8 (66.7)	3 (75.0)	5 (62.5)
Regional anesthesia	4 (33.3)	1 (25.0)	3 (37.5)
Anesthesia duration (min)	119 ± 44	122 ± 24	117 ± 53
Mechanical ventilation	8 (66.7)	3 (75.0)	5 (62.5)
Ventilation duration (min)	1324 ± 3250	3208 ± 5328	194 ± 173
Mean peak airway pressure (mmHg)	23 ± 4	22 ± 6	23 ± 3
Respiratory rate	14 ± 4	17 ± 6	13 ± 2
Back to ICU without extubation	2 (16.7)	1 (25.0)	1 (12.5)

The results are presented as the means ± standard deviations (SD) or n (%)

*PP group*, patients developed postoperative pneumothorax; *nPP group*, patients did not develop postoperative pneumothorax; *TSC*, tuberous sclerosis complex; *LAM*, lymphangioleiomyomatosis; *CT*, computerized tomography; *VEGF-D*, vascular endothelial growth factor-D; *FEV*_*1*_, forced expiratory volume in 1 s; *DLco*, diffusing capacity for carbon monoxide; *6MWD*, 6-min walking distance; *PaO*_*2*_, partial pressure of oxygen in arterial blood; *SpO*_*2*_, room air pulse oxygen saturation; *PTX*, pneumothorax; *mTOR*, mammalian target of rapamycin; *VATS*, video-assisted thoracoscopic; *ASA, American Society of Anesthesiologists; ICU*, intensive care unit

^*a*^ Sample size for CT grade was 11, with 3 in PP group and 8 in nPP group. CT grade is classified based on the proportion of cystic lesions in total lung. I: < 1/3, II: 1/3 – 2/3, III: > 2/3

^*b*^ Sample size for VEGF-D was 10, with 3 in PP group and 7 in nPP group

^*c*^ Sample size for FEV_1_%Pred was 9, with 2 in PP group and 7 in nPP group

^*d*^ Sample size for DLco%Pred was 9, with 2 in PP group and 7 in nPP group

^*e*^ Sample size for 6MWD was 11, with 3 in PP group and 8 in nPP group

As for intraoperative variables, however, there was little difference between the two groups in whether patients underwent pulmonary surgery, received general or regional anesthesia, and experienced mechanical ventilation. The parameters of mechanical ventilation (mean peak airway pressure and respiratory rate) and whether intensive care unit (ICU) without extubation did not different either. The duration of mechanical ventilation in PP group patients was significantly prolonged (*P* = 0.002), which may be related to one patient who developed postoperative PTX during ICU admission before extubation and continued mechanical ventilation for several days (Table 1, Suppl 1).

For the four patients developing postoperative PTX, detailed information is listed in Table 2. As we can see, the postoperative PTXs all occurred in more than one week after the surgery - postoperative days (POD) 10, POD 20, POD 13 and POD 8, respectively. Most patients showed II to III of CT grades and poor pulmonary ventilation and diffusion capability. Three patients had preoperative PTX and even recurrent PTXs, and no patients had been treated with mTOR inhibitors before surgery. Two patients underwent pulmonary surgery, and another two underwent cesarean section. Three patients received general anesthesia and experienced mechanical ventilation during the surgery, with two extubated successfully after surgery and one back to ICU without extubation. One patient received regional anesthesia and did not experience intraoperative mechanical ventilation.

**Table 2:** Clinical data for four patients with lymphangioleiomyomatosis who experienced postoperative pneumothorax

Characteristics	Case 1	Case 2	Case 3	Case 4
Age (y)	34	49	30	33
Diagnosis	Sporadic-LAM	TSC-LAM	Sporadic-LAM	Sporadic-LAM
**Preoperative variables**				
CT grade	/	II	III	III
VEGF-D (pg/mL)	/	679	1734	1211
FEV_1_/Pred (%)	/	/	64	75
DLco/Pred (%)	/	/	25	47
6MWD (m)	/	590	410	472
PaO_2_ (mmHg)	73	65.5	62	85
SpO_2_ (%)	87	100	90	95
Number of preoperative PTX episodes	7	2	1	0
History of pleurodesis	No	No	No	No
Menstruation status	Pre-menopausal	Peri-menopausal	Pre-menopausal	Pre-menopausal
Current pregnancy	No	No	Yes	Yes
Preoperative mTOR inhibitors therapy	No	No	No	No
**Intraoperative variables**				
Surgical type	VATS lobectomy	VATS pleurodesis	Cesarean section	Cesarean section
Surgery duration (min)	118	75	60	63
ASA physical status	3	2	3	4
Anesthetic technology	GA	GA	CSEA	GA
Anesthesia duration (min)	153	125	115	95
Mechanical ventilation	Yes	Yes	No	Yes
Ventilation duration (min)	153	112	/	9360
Mean peak airway pressure (mmHg)	19	17	/	29
Respiratory rate	22	11	/	18
Extubation	Yes	Yes	/	No
Back to ICU	Yes	No	Yes	Yes
**Date of postoperative PTX**	POD 10	POD 20	POD 13	POD 8

*CT*, computerized tomography; *VEGF-D*, vascular endothelial growth factor-D; *FEV*_*1*_, forced expiratory volume in 1 s; *DLco*, diffusing capacity for carbon monoxide; *6MWD*, 6-min walking distance; *PaO*_*2*_, partial pressure of oxygen in arterial blood; *SpO*_*2*_, room air pulse oxygen saturation; *PTX*, pneumothorax; *mTOR*, mammalian target of rapamycin; *ASA, American Society of Anesthesiologists; ICU*, intensive care unit; *LAM*, lymphangioleiomyomatosis; *TSC*, tuberous sclerosis complex; *VATS*, video-assisted thoracoscopic; *GA*, general anesthesia; *CSEA*, combined spinal and epidural anesthesia; *POD*, postoperative days

## Discussion

From our results, preoperative factors seem more important for the risk evaluation of LAM. Patients with higher CT grade, poorer pulmonary function (both ventilation and diffusion function), and a history of preoperative PTX might be more likely to develop postoperative PTX, consistent with previous research [9]. However, the intraoperative factors, especially mechanical ventilation that we usually concerned, did not show obvious effect on postoperative PTX, as we imagined. These findings might indicate that for patients with well-controlled preoperative conditions, intraoperative mechanical ventilation may not increase the risk of postoperative PTX. Higher PaO_2_ and SpO_2_ on room air may also convey a safer message for mechanical ventilation if patients are unsuitable for pulmonary function testing. Consistent with the potential protective effect of mTOR inhibitors in LAM patients [10], patients with preoperative mTOR inhibitor therapy in our study did not experience postoperative PTX even under mechanical ventilation.

According to previous research, appropriate protective ventilation strategies, such as lower positive end expiratory pressure, no recruitment maneuvers and nitrous oxide, and pressure-controlled or pressure-regulated volume-controlled ventilation, are recommended in general anesthesia for LAM patients to prevent PTX [6]. However, our results suggest that those with poor preoperative conditions may still be at high risk of postoperative PTX regardless of whether mechanical ventilation is used and what ventilation strategies are chosen during surgery. For such patients, controlling the progress of disease and improving pulmonary function before surgery might be more essential.

In our study, half of the patients underwent pulmonary surgery. Since some postoperative PTXs are related to pulmonary procedure itself, this might greatly bias the risk of postoperative PTX. However, postoperative PTX in this study all occurred in more than one week after the surgery, which were more likely to be disease-related rather than procedure-related. On the other hand, the incidence of postoperative PTX was quite the same in patients receiving pulmonary and non-pulmonary surgery (33.3%), and the proportion of patients undergoing pulmonary and non-pulmonary surgery in PP group was also the same (50.0%). In addition, according to the information shown in Suppl 2, patients received lobectomy or pleurodesis mainly because of recurrent spontaneous PTX or in order to make a definite diagnosis, and those who with good preoperative conditions did not develop postoperative PTX even undergoing pulmonary surgery. Therefore, the bias of pulmonary surgery on the risk of postoperative PTX may be not large, but this inference may not be generalized in a larger population considering the limited sample size.

Although we included as many surgical patients with LAM as possible in this study, the sample size was still small due to the extremely low prevalence of LAM (3.4 to 7.8 per million women) [11], and thus limited the power of statistical analysis. Despite no specific conclusion could be drawn from these 12 patients, our results indeed help clinicians reconsider the perioperative management of LAM patients. Whether intraoperative mechanical ventilation could increase the risk of PTX for patients with LAM might depend more on patients’ preoperative conditions, especially the CT grade, pulmonary function, history of spontaneous PTX, and mTOR inhibitor therapy. Further studies with larger sample sizes and including more detailed mechanical ventilation parameters are needed to verify our findings and discover more information about the perioperative management of patients with LAM.

### Electronic supplementary material

Below is the link to the electronic supplementary material.

Supplementary Material 1

Supplementary Material 2

## Data Availability

The datasets generated and/or analyzed during the current study are not publicly available due to IRB restrictions. Data can be made available from the corresponding author on reasonable request and with the appropriate IRB approvals.

## Abbreviations

ASA: American Society of Anesthesiologists
CSEA: Combined spinal and epidural anesthesia
CT: Computerized tomography
DLco: Diffusing capacity for carbon monoxide
FEV_1_: Forced expiratory volume in 1 s
GA: General anesthesia
ICU: Intensive care unit
LAM: Lymphangioleiomyomatosis
mTOR: Mammalian target of rapamycin
nPP group: Patients who did not develop postoperative pneumothorax
PaO_2_: Partial pressure of oxygen in arterial blood
POD: Postoperative days
PP group: Patients who developed postoperative pneumothorax
PTX: Pneumothorax
SpO_2_: Room air pulse oxygen saturation
TSC: Tuberous sclerosis complex
VATS: Video-assisted thoracoscopic
VEGF-D: Vascular endothelial growth factor-D
6MWD: 6-min walking distance
