# Supplementary material for: Intraoperative mechanical ventilation and incidence of pneumothorax in lymphangioleiomyomatosis
Source: Orphanet J Rare Dis. 2024 Mar 23;19:133. doi: 10.1186/s13023-024-03117-w (PMC10960991; doi:10.1186/s13023-024-03117-w)
Supplement: Supplementary file 2 — Supplementary Material 2 [file 13023_2024_3117_MOESM2_ESM.docx]

**Suppl 1 Descriptive statistical analysis of surgical patients with lymphangioleiomyomatosis**

| **Variables** | **All patients**  **(n=12)** | **PP group**  **(n=4)** | **nPP group**  **(n=8)** | ***P* value** |
| --- | --- | --- | --- | --- |
| Age (y) | 41±10 | 37±9 | 43±11 | 0.505 |
| Diagnosis |  |  |  |  |
| TSC-LAM | 1 (8.3) | 1 (25.0) | 0 (0.0) | Reference |
| Sporadic-LAM | 11 (91.7) | 3 (75.0) | 8 (100.0) | 0.140 |
|  |  |  |  |  |
| **Preoperative variables** |  |  |  |  |
| CT grade*^a^* |  |  |  |  |
| I~II | 6 (54.6) | 1 (33.3) | 5 (62.5) | Reference |
| III | 5 (45.5) | 2 (66.7) | 3 (37.5) | 0.387 |
| VEGF-D (pg/mL)*^b^* | 1145±832 | 1208±528 | 1119±971 | 0.124 |
| FEV_1_%pred (%)*^c^* | 77.2±25.8 | 69.5±7.8 | 79.1±28.7 | 0.200 |
| DLco%pred (%)*^d^* | 60.2±29.7 | 36.0±15.6 | 66.3±29.9 | 0.232 |
| 6MWD (m)*^e^* | 501±69 | 491±91 | 505±66 | 0.432 |
| PaO_2_ (mmHg) | 80.1±13.5 | 71.4±10.2 | 84.5±13.3 | 0.250 |
| SpO_2_ (%) | 96±4 | 93±6 | 98±2 | 0.008 |
| Preoperative PTX | 6 (50.0) | 3 (75.0) | 3 (37.5) | 0.221 |
| History of pleurodesis | 2 (16.7) | 0 (0.0) | 2 (25.0) | 0.273 |
| Menstruation status |  |  |  | 0.223 |
| Pre-menopausal | 9 (75.0) | 3 (75.0) | 6 (75.0) |  |
| Peri-menopausal | 1(8.3) | 1 (25.0) | 0 (0.0) |  |
| Post-menopausal | 2 (16.7) | 0 (0.0) | 2 (25.0) |  |
| Current pregnancy | 4 (33.3) | 2 (50.0) | 2 (25.0) | 0.386 |
| Preoperative mTOR inhibitors therapy | 3 (25.0) | 0 (0.0) | 3 (37.5) | 0.157 |
|  |  |  |  |  |
| **Intraoperative variables** |  |  |  |  |
| Surgical type |  |  |  |  |
| Non-pulmonary surgery | 6 (50.0) | 2 (50.0) | 4 (50.0) | Reference |
| VATS lobectomy or pleurodesis | 6 (50.0) | 2 (50.0) | 4 (50.0) | 1.000 |
| Surgery duration (min) | 79±40 | 79±28 | 79±47 | 0.666 |
| Timing of surgery |  |  |  |  |
| Elective | 11 (91.7) | 3 (75.0) | 8 (100.0) | Reference |
| Emergent | 1 (8.3) | 1 (25.0) | 0 (0.0) | 0.140 |
| ASA physical status |  |  |  |  |
| 1～2 | 7 (58.3) | 1 (25.0) | 6 (75.0) | Reference |
| 3～4 | 5 (41.7) | 3 (25.0) | 2 (50.0) | 0.098 |
| Anesthetic technology |  |  |  |  |
| Regional anesthesia | 4 (33.3) | 1 (25.0) | 3 (37.5) | Reference |
| General anesthesia | 8 (66.7) | 3 (75.0) | 5 (62.5) | 0.665 |
| Anesthesia duration (min) | 119±44 | 122±24 | 117±53 | 0.367 |
| Mechanical ventilation | 8 (66.7) | 3 (75.0) | 5 (62.5) | 0.665 |
| Ventilation duration (min) | 1324±3250 | 3208±5328 | 194±173 | 0.002 |
| Mean peak airway pressure (mmHg) | 23±4 | 22±6 | 23±3 | 0.071 |
| Respiratory rate | 14±4 | 17±6 | 13±2 | 0.095 |
| Back to ICU without extubation | 2 (16.7) | 1 (25.0) | 1 (12.5) | 0.495 |

The results are presented as the means ± standard deviations (SD) or n (%).

*PP group,* patients developed postoperative pneumothorax; *nPP group,* patients did not develop postoperative pneumothorax; *TSC,* tuberous sclerosis complex; *LAM,* lymphangioleiomyomatosis; *CT,* computerized tomography; *VEGF-D,* vascular endothelial growth factor-D; *FEV_1_,* forced expiratory volume in 1 s; *DLco,* diffusing capacity for carbon monoxide; *6MWD,* 6-min walking distance; *PaO_2_,* partial pressure of oxygen in arterial blood; *SpO_2_,* room air pulse oxygen saturation; *PTX,* pneumothorax; *mTOR,* mammalian target of rapamycin; *VATS,* video-assisted thoracoscopic; *ICU,* intensive care unit.

*^a^* Sample size for CT grade was 11, with 3 in PP group and 8 in nPP group. CT grade is classified based on the proportion of cystic lesions in total lung. I: < 1/3, II: 1/3 ~ 2/3, III: > 2/3.

*^b^* Sample size for VEGF-D was 10, with 3 in PP group and 7 in nPP group.

*^c^* Sample size for FEV_1_%Pred was 9, with 2 in PP group and 7 in nPP group.

*^d^* Sample size for DLco%Pred was 9, with 2 in PP group and 7 in nPP group.

*^e^* Sample size for 6MWD was 11, with 3 in PP group and 8 in nPP group.

**Suppl 2 Clinical data for lymphangioleiomyomatosis patients receiving pulmonary surgery**

| **Characteristics** | **Case 1** | **Case 2** | **Case 3** | **Case 4** | **Case 5** | **Case 6** |
| --- | --- | --- | --- | --- | --- | --- |
| Age (y) | 34 | 39 | 49 | 48 | 62 | 38 |
| Diagnosis | Sporadic-LAM | Sporadic-LAM | TSC-LAM | Sporadic-LAM | Sporadic-LAM | Sporadic-LAM |
|  |  |  |  |  |  |  |
| **Preoperative variables** |  |  |  |  |  |  |
| CT grade | / | 1 | 2 | 1 | 1 | 1 |
| VEGF-D (pg/mL) | / | 260 | 679 | 666 | / | 354 |
| FEV_1_/Pred (%) | / | 98 | / | 71.1 | 122 | 106 |
| DLco/Pred (%) | / | 84 | / | 80 | 106 | 92 |
| 6MWD (m) | / | 510 | 590 | 490 | 505 | 525 |
| PaO_2_ (mmHg) | 73 | 98 | 65.5 | 95.8 | 86.7 | 92.2 |
| SpO_2_ (%) | 87 | 100 | 100 | 100 | 98 | 98 |
| Number of preoperative PTX episodes | 7 | 0 | 2 | 3 | 0 | 0 |
| History of pleurodesis | 0 | 0 | 0 | 0 | 0 | 0 |
| Menstruation status | Pre-menopausal | Peri-menopausal | Peri-menopausal | Pre-menopausal | Post-menopausal | Pre-menopausal |
| Current pregnancy | No | No | No | No | No | No |
| Perioperative mTOR inhibitors therapy | No | No | No | Yes | No | No |
|  |  |  |  |  |  |  |
| **Intraoperative variables** |  |  |  |  |  |  |
| Surgical type | VATS lobectomy | VATS lobectomy | VATS pleurodesis | VATS pleurodesis | VATS lobectomy | VATS lobectomy |
| Surgical indications | Recurrent spontaneous PTX | To make a definite diagnosis | Recurrent spontaneous PTX | Recurrent spontaneous PTX | To make a definite diagnosis | To make a definite diagnosis |
| Surgery duration (min) | 118 | 40 | 75 | 73 | 74 | 65 |
| ASA physical status | 3 | 1 | 2 | 2 | 1 | 2 |
| Anesthetic technology | GA | GA | GA | GA | GA | GA |
| Anesthesia duration (min) | 153 | 80 | 125 | 102 | 109 | 102 |
| Mechanical ventilation | Yes | Yes | Yes | Yes | Yes | Yes |
| Ventilation duration (min) | 153 | 60 | 112 | 94 | 103 | 480 |
| Mean peak airway pressure (mmHg) | 19 | 20 | 17 | 25 | 23 | 27 |
| Respiratory rate | 22 | 12 | 11 | 14 | 15 | 10 |
| Extubation | Yes | Yes | Yes | Yes | Yes | No |
| Back to ICU | Yes | No | No | No | Yes | Yes |
|  |  |  |  |  |  |  |
| **PTX within 30 days after surgery** | Yes | No | Yes | No | No | No |

*CT,* computerized tomography; *VEGF-D,* vascular endothelial growth factor-D; *FEV_1_,* forced expiratory volume in 1 s; *DLco,* diffusing capacity for carbon monoxide; *6MWD,* 6-min walking distance; *PaO_2_,* partial pressure of oxygen in arterial blood; *SpO_2_,* room air pulse oxygen saturation; *PTX,* pneumothorax; *mTOR,* mammalian target of rapamycin; *ASA, American Society of Anesthesiologists; ICU,* intensive care unit; *LAM,* lymphangioleiomyomatosis; *TSC,* tuberous sclerosis complex; *VATS,* video-assisted thoracoscopic; *GA,* general anesthesia.
